# Motor learning enhanced by combined motor imagery and noninvasive brain stimulation is associated with reduced short‐interval intracortical inhibition

**DOI:** 10.1002/brb3.1252

**Published:** 2019-03-18

**Authors:** Hai‐Jiang Meng, Na Cao, Yi‐Tong Lin, Ke Liu, Jian Zhang​, Yan‐Ling Pi

**Affiliations:** ^1^ School of Sports Anqing Normal University Anqing China; ^2^ School of Kinesiology Shanghai University of Sport Shanghai China; ^3^ Shanghai Punan Hospital of Pudong New District Shanghai China

**Keywords:** motor cortex, motor evoked potential, paired associative stimulation, transcranial magnetic stimulation

## Abstract

**Background:**

Motor imagery (MI) improves motor skill learning, which is further enhanced when MI is paired with primary motor cortex transcranial brain stimulation or with electrical stimulation of the peripheral median nerve. Applying both stimulation types (here with 25 ms intervals) is called paired associative stimulation (PAS25). The final primary motor cortex output is determined by combined excitatory and intracortical inhibitory circuits, and reducing the latter is associated with enhanced synaptic transmission and efficacy. Indeed, short‐interval intracortical inhibition (SICI) inhibits motor evoked potentials (MEPs), and motor learning has been associated with decreased SICI and increased cortical excitability. Here, we investigated whether cortical excitability and SICI are altered by PAS25 applied after MI‐induced modulation of motor learning.

**Methods:**

Peak acceleration of a hand‐grasping movement and MEPs and SICI were measured before and after MI alone, PAS25 alone, and MI followed by PAS25 in 16 healthy participants to evaluate changes in motor learning, corticospinal excitability, and intracortical inhibition.

**Results:**

After PAS25 alone, MEP amplitude increased while peak acceleration was unchanged. However, PAS25 applied following MI not only significantly enhanced both peak acceleration (*p* = 0.011) and MEP amplitude (*p* = 0.004) but also decreased SICI (*p* = 0.011). Moreover, we found that this decrease in SICI was significantly correlated with both the peak acceleration (*r* = 0.49, *p* = 0.029) and the MEP amplitude (*r* = 0.56, *p* = 0.013).

**Conclusions:**

These results indicate that brain function altered by PAS25 of the motor cortex enhances MI‐induced motor learning and corticospinal excitability and decreases SICI, suggesting that SICI underlies, at least in part, PAS25 modulation of motor learning.

## 1 INTRODUCTION

Synaptic plasticity, which is critical for learning and memory, can be induced within the human cortex using noninvasive brain stimulation (Huang, Edwards, Rounis, Bhatia, & Rothwell, 2005; Stefan, Kunesch, Cohen, Benecke, & Classen, 2000). Recent studies have used noninvasive brain stimulation to deliberately change human brain function and to assist in the establishment of new brain connections (i.e., synaptic plasticity) to thus improve motor performance in motor skill learning. The effectiveness of noninvasive brain stimulation on motor skill learning indicates that such stimulation protocols have been appropriately developed to alter synaptic transmission and efficacy (Cogiamanian, Marceglia, Ardolino, Barbieri, & Priori, 2007; Okano et al., 2015; Polania, Nitsche, & Ruff, 2018). Synaptic transmission and efficacy altered by noninvasive brain stimulation are significantly enhanced by reducing intracortical inhibition in the primary motor cortex (Cash, Murakami, Chen, Thickbroom, & Ziemann, 2016; Stefan, Kunesch, Benecke, Cohen, & Classen, 2002). Although several experiments have provided direct evidence for this increased synaptic transmission and efficacy, the mechanisms undergirding the intracortical inhibition and corticospinal excitability changes when noninvasive brain stimulation promotes performance of motor skill learning remain unclear.

Motor imagery (MI) without sensory input and external motor output is a useful approach for enhancing motor skill learning. Studies in human have shown that although the beneficial effects of short‐term MI training on learning are weaker than motor practice (Bonassi et al., 2017), the effects of MI can be enhanced when paired with simultaneous anodal transcranial direct current stimulation over the primary motor cortex or simultaneous electrical stimulation applied to the median nerve at the wrist (Bonassi et al., 2017; Saimpont et al., 2016). Here, we tested whether the beneficial effects of short‐term MI are enhanced by applying combined peripheral median nerve electrical stimulation with transcranial magnetic stimulation (TMS) of the motor cortex after MI. Applying these two noninvasive brain stimulation techniques with specific intervals between the two stimulation paradigms is termed as paired associative stimulation (PAS). The use of PAS interstimulus intervals of 25 ms (PAS25) induces long‐lasting enhancement of excitability in the human motor cortex (duration >30 min), termed long‐term potentiation (LTP)—like (Stefan et al., 2002, 2000). Human TMS studies have shown that the neural circuits activated by PAS25 are the same as those activated by motor learning (Sale & Mattingley, 2013; Ziemann, Ilic, Pauli, Meintzschel, & Ruge, 2004). Therefore, we hypothesized that application of PAS25 in participants after they performed MI would improve their learning of a motor skill.

The balance and interactions between intracortical inhibitory and excitatory circuits determine the final output from the primary motor cortex (Ni et al., 2011). Whereas a single‐pulse TMS protocol can be used to investigate the corticospinal excitability, paired‐pulse TMS protocols can be used to study intracortical neural circuits. Short‐interval intracortical inhibition (SICI), a common and well‐investigated intracortical inhibitory phenomenon (Dai et al., 2016; Ni, Muller‐Dahlhaus, Chen, & Ziemann, 2011), can be elicited when a subthreshold conditioning stimulation suppresses a subsequent suprathreshold test stimulation (at interstimulus intervals of 1–5 ms) to inhibit the subsequent motor evoked potential (Ni, Gunraj, Kailey, Cash, & Chen, 2014). Pharmacological studies have suggested that SICI is mediated by type A gamma‐aminobutyric acid (GABA_A_) receptors (Di Lazzaro et al., 2005). Studies of the human motor cortex using TMS have reported increased cortical excitability and decreased SICI after motor learning (Leung, Rantalainen, Teo, & Kidgell, 2015; Rosenkranz, Kacar, & Rothwell, 2007), suggesting that GABA_A_ plays an important role in human motor learning (Kolasinski & Hinson, 2018).

In this study, we examined whether cortical excitability and SICI are also altered after brain‐stimulated modulation of the motor learning enhanced by MI. We hypothesized that cortical excitability would be increased when PAS25 following MI improved the learning induced by MI and that this increased cortical excitability would be accompanied by decreased SICI. Such a finding would suggest that PAS25 plays an important role in the enforcement of the motor learning produced by MI and that changing brain function using noninvasive brain stimulation technology may be an effective strategy for improving motor learning. If true, this strategy may not only provide athletes with a competitive advantage but also offer a potential therapeutic tool for rehabilitation in patients with stroke.

## 2 METHODS

### 2.1 Participants

Sixteen healthy participants aged 18–28 years (mean age: 22.94 ± 2.72 years; eight women and eight men) were included in this study. All participants were confirmed to be right‐handed using the Oldfield Handedness Inventory (Oldfield, 1971). No participant had a history of neurological, psychiatric, or other medical disorder, nor did anyone exhibit any contraindication to TMS. All participants gave their written informed consent. All experiments were approved by the Human Research Ethics Committee of the Shanghai University of Sports and were conducted in accordance with the Declaration of Helsinki.

### 2.2 Experimental design

**Figure 1:**
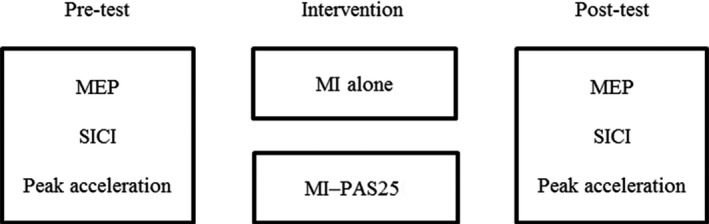
Experimental design. Two different interventional protocols were compared in the experiment. The interventional protocols were MI alone and MI followed by PAS25 (MI‐PAS25). Measurements were taken before and immediately after each interventional protocol. The measurements were MEP amplitude, SICI, and peak acceleration. PAS25, pair associative stimulation (PAS) with 25 ms interstimulus intervals between median nerve stimulation and transcranial magnetic stimulation; MI, motor imagery; MEP, motor evoked potential; SICI, short‐interval intracortical inhibition

The experiment was conducted using a repeated measures design. We performed the main experiment to investigate the effects of PAS25 applied after MI on MI‐induced motor learning, corticospinal excitability, and SICI. Two experimental sessions were conducted for each participant: MI alone, and MI followed by PAS25 (MI‐PAS25). The order of the two experimental sessions was counterbalanced among the participants. There were at least 2 weeks between each experimental session to avoid interference effects. Measurements were performed before and immediately after each intervention (Figure 1).

Ten participants also performed a control experiment to verify that PAS25 alone could induce changes of corticospinal excitability in the primary motor cortex. The experimental procedures were the same as those for the main experiment.

### 2.3 Motor imagery

Participants were comfortably seated in a chair with a fixed headrest. They were required to repeatedly imagine a right‐hand grasping movement using all fingers in a first person perspective, with the imagined action self‐paced. A sensory stimulation cue applied at the left wrist (stimulus intensity of two times the sensory threshold), rhythmically delivered at a frequency of 0.1 Hz by a Digitimer DS7A (Digitimer Ltd.; Hertfordshire, UK) constant current stimulator (0.2 ms square‐wave pulses) with standard bar electrodes (cathode proximal), was given as the motor preparation. Participants closed their eyes before starting the imagery and opened them at the end. Participants completed one imagined movement within 10 s, for a total of 90 times during 15 min.

In order to standardize the task among participants, the grasping movement and the precise instructions related to the kind of grasp requested were displayed by videos. One week before the formal TMS experiment, participants were trained for a few hours to mentally simulate the action, trying to “feel the same as during actual execution” (kinesthetic imagery).

To confirm that participants were able to form mental images with sufficient vividness, they completed a Movement Imagery Questionnaire‐Revised (MIQ‐R) before each behavioral intervention (Fourkas, Bonavolonta, Avenanti, & Aglioti, 2008). The MIQ‐R included four kinesthetic items and four visual items and used a 7‐point scale, with a score of 7 corresponding to the greatest difficulty during the imagery.

A self‐evaluation MI questionnaire of four kinesthetic and four visual items further measured the difficulty (on a scale of 1, “very easy,” to 7, “very difficult”) of forming kinesthetic and visual images of grasping movements after each behavioral intervention. The four kinesthetic items were difficulty of kinesthetic imagery, sequence of muscle contraction, muscle tension, and grasping force during imagery. The four visual items were difficulty of visual imagery, clarity of all fingers in the right hand, clarity of grasping action, and clarity of all fingers expanding.

### 2.4 PAS25

PAS25 consisted of electrical stimuli applied to the right median nerve at the wrist followed by TMS applied over the left primary motor cortex at an interstimulus interval of 25 ms. During a period of 15 min, 90 pairs of stimulations were delivered with a frequency of 0.1 Hz. Median nerve stimulation was applied to the right wrist using a Digitimer DS7A constant current stimulator (0.2 ms square‐wave pulses) with standard bar electrodes (cathode proximal). The stimulus intensity was three times the sensory threshold. The TMS intensity was set at 1 mV for each participant (Ni et al., 2014).

### 2.5 Behavioral measurement

We quantified the change in learning by measuring the mean peak acceleration of an externally paced, fastest possible, right‐hand grasping movement immediately before and after each intervention. Acceleration of the grasping movement was measured using a three‐dimensional (3D) infrared motion‐tracking system (Vicon 612, Vicon Motion Systems Ltd.; Oxford, UK), with Workstation software version 5.1. The infrared high‐speed cameras (MX13, with a sampling frequency of 300 Hz) were set up for an experimental shooting range of approximately 9 × 2 × 2 m^3^. The cameras were connected to a Vicon workstation system and calibrated for this 3D shooting space. When grasping, the thumb joints deform more than the index finger joints, and a marker ball will more easily fall off the thumb. Therefore, one marker ball, with a diameter of 14 mm, was placed at the second joint and another at the third joint of the participant's right index finger.

The participants stood upright within the shooting range. The right elbow was bent 90° to the right side of the body. When the participants heard the previously agreed on start word, they made a rapid grasping movement with the right hand. Participants were continuously encouraged by the experimenter to make grasping movements that led to an acceleration in the direction of flexion as fast as possible.

Each participant successfully provided at least two sets of data before and after each intervention. Kinematic data were collected by the experimenter and input into a laboratory computer for off‐line analysis. Visual 3D analysis software, version 3.9.1 (C‐Motion, United States), was used to perform preliminary processing analysis of the behavioral data.

### 2.6 Corticospinal excitability and SICI measurements

Measurements included resting motor threshold, MEP amplitude, and SICI. Resting motor threshold for the right abductor pollicis brevis (APB) muscle was defined as the lowest TMS intensity needed to generate MEPs of >50 µV in at least 5 of 10 trials when the muscle was completely relaxed. The MEP amplitude was measured with a “1‐mV TMS intensity” determined before each intervention. SICI at interstimulus intervals of 2 ms was tested using a paired‐pulse TMS. The test pulse intensity was set at “1 mV” adjusted as needed at each time point, and the conditioning pulse intensity was set at 70% of the resting motor threshold. This 1‐mV intensity was defined as the lowest TMS intensity needed to generate MEPs of >1 mV in at least 5 of 10 trials in the APB muscle when the muscle was completely relaxed.

Transcranial magnetic stimulation was applied to the left primary motor cortex with a figure‐eight‐shaped coil (outside diameter of each loop was 9.5 cm) connected to a Magstim 200 stimulator (Magstim; Whitland, Dyfed, UK). The coil was held tangentially to the skull with the handle (approximately perpendicular to the central sulcus) pointing backward and laterally at an angle of 45° to the sagittal plane. Monophasic pulses, which produced posterior–anteriorly directed current in the brain, were used to deliver TMS. For the paired‐pulse experiment, two Magstim 200 stimulators were connected to the same coil through a BISTIM module. The right APB muscle was selected as the target muscle because it is innervated by the median nerve. The optimal scalp position for inducing MEPs in the right APB muscle was found by moving the coil in steps of 1 cm until the largest MEPs were found; this position was marked with a pen and was considered the motor hot spot.

Surface electromyograms were recorded from the right APB and first dorsal interosseous (FDI) muscles with 9‐mm‐diameter Ag‐AgCl surface electrodes. The active electrode was placed on the muscle belly, and the reference electrode was placed on the metacarpophalangeal joint of the finger. A ground electrode was placed on the right wrist. The signal was amplified (1,000×), band‐pass filtered (20 Hz to 2.5 kHz; Intronix Technologies Model 2024F), digitized at a rate of 5 kHz by an analog‐to‐digital interface (Micro1401, Cambridge Electronics Design; Cambridge, UK), and stored in a computer for off‐line analysis.

### 2.7 Data and statistical analyses

The MIQ‐R scores for the various experimental conditions were compared using the nonparametric Wilcoxon test. Paired *t* tests were used to assess the effects of PAS25 alone on peak acceleration and on MEP amplitude. A two‐way repeated measures analysis of variance (ANOVA) was used to assess the effects of the interventional protocol and time on the different measurements. *Post hoc* paired *t *tests with Bonferroni corrections for multiple comparisons were used to determine at which time point the measurement was different among the various interventional protocols when the results of the ANOVA showed significant main effects or interactions. The relationship between corticospinal excitability, peak acceleration, and intracortical inhibition was assessed using Pearson's correlation test.

The MEP amplitudes were measured peak to peak. For SICI, The paired‐pulse‐induced MEP amplitude was expressed as a percentage of the mean MEP amplitude of test stimulus alone. Values <100% indicate inhibition and values >100% indicate facilitation. SPSS software, version 17.0 (IBM; Armonk, NY) was used for all statistical analyses. The threshold for statistical significance was *p* < 0.05. Values are reported as the mean ± *SE*.

## 3 RESULTS

### 3.1 Effects of PAS25 alone on peak acceleration and MEP amplitude

**Figure 2:**
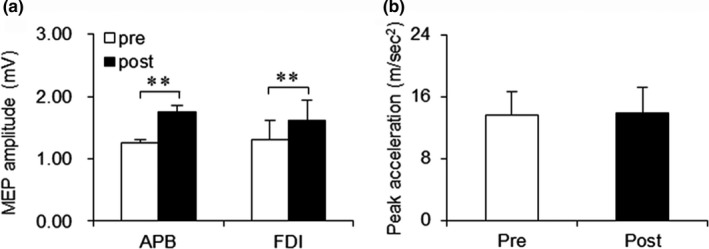
Effects of PAS25 alone on MEP amplitude and peak acceleration. (a) MEP amplitude measured immediately after PAS25 is significantly greater than that measured before PAS25 in the APB and FDI muscles (**p < 0.01). (b) PAS25 does not change peak acceleration before (Pre) and after (Post) application of PAS25 alone

Figure 2 displays the effects of PAS25 alone on peak acceleration and MEP amplitude in the control experiment. Paired *t *tests were used to compare the results before and after PAS25 alone. The results of these tests indicated that PAS25 did not significantly alter the peak acceleration of the fastest possible grasping movement (before PAS25, 13.13 ± 1.45 m/sec^2^ vs. after PAS25, 12.53 ± 2.22 m/sec^2^; *t*_9_ = 0.58, *p* = 0.575).

By contrast, comparison of the time points immediately before and after PAS25 indicated a significant increase in MEP amplitude in both the target APB muscle (MEP amplitude before PAS25, 1.25 ± 0.05 mV vs. MEP amplitude after PAS25, 1.75 ± 0.11 mV;* t*_9_ = −4.69, *p = *0.001) and nontarget FDI muscle (MEP amplitude before PAS25, 1.31 ± 0.31 mV vs. MEP amplitude after PAS25, 1.61 ± 0.33 mV; *t*_9_ = −4.03, *p = *0.003).

### 3.2 Effects of PAS25 on peak acceleration after MI

#### 3.2.1 MI ability

Table 1 gives the mean MI ability and clarity (i.e., ease with which the imagery is experienced) measures scores of all participants after MI‐PAS25 and after MI alone. There was no difference in MIQ‐R scores for either kinesthetic (*z *= −1.03, *p = *0.304) or visual (*z *= −1.08, *p = *0.278) items, or for self‐evaluation MI scores on either kinesthetic (*z *= −1.52, *p = *0.128) or visual (*z *= −0.36, *p = *0.718) items between the two experimental conditions. These results indicated that the MI ability and clarity of participants were not significantly different under the two interventional protocols. In addition, their scores indicated that all participants possessed good MI ability and could clearly visualize the images.

**Table 1:** Evaluation of motor imagery ability under MI‐PAS25 and MI conditions

	MI‐PAS25	MI
Movement imagery questionnaire		
Kinesthetic items	8.00 ± 0.67	7.56 ± 0.64
Visual items	7.50 ± 0.61	6.81 ± 0.53
Self‐evaluation questionnaire		
Kinesthetic items	11.63 ± 0.47	10.63 ± 0.72
Visual items	8.19 ± 0.68	7.94 ± 0.56

Note

PAS25: pair associative stimulation (PAS) with 25 ms interstimulus intervals between median nerve stimulation and transcranial magnetic stimulation; MI: motor imagery.

#### 3.2.2 Peak acceleration

**Figure 3:**
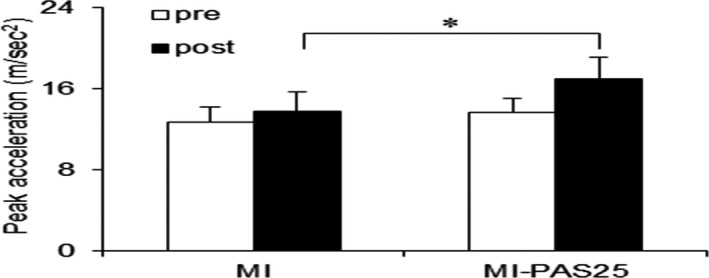
Peak acceleration before and after motor imagery (MI) alone or MI‐PAS25. Peak acceleration measured immediately after MI‐PAS25 (post) is significantly greater relative to that measured after MI alone (Post) (*p < 0.05)

Figure 3 shows the modulatory effects of PAS25 on motor learning after MI. The main effects of time (*F*_(1,15)_ = 7.04, *p* = 0.018) and interventional protocol (*F*_(1,15)_ = 5.21, *p* = 0.037) as well as their interaction (*F*_(1,15)_ = 8.56, *p* = 0.01) were found to be significant in a repeated measures ANOVA. *Post hoc *tests indicated that the peak acceleration of the grasping movement performed after MI‐PAS25 was significantly faster than that performed after MI alone (*p* = 0.011).

### 3.3 Effects of PAS25 on MEP amplitude and SICI after MI

#### 3.3.1 MEP amplitude

**Figure 4:**
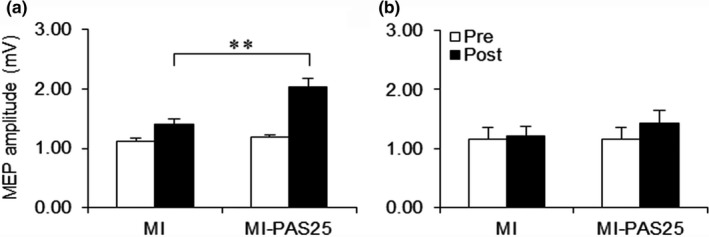
MEP amplitude before and after motor imagery (MI) alone or MI‐PAS25. (a) MEP amplitude measured immediately after MI‐PAS25 (Post) is significantly greater relative to that after MI alone (Post) in the APB muscle (**p < 0.01). (b) There is no difference in MEP amplitude between MI and MI‐PAS25 in the FDI muscle

For the target APB muscle (Figure 4a), the results of a repeated measures ANOVA indicated significant main effects of time (*F*_(1,15)_ = 56.35, *p < *0.001) and interventional protocol (*F*_(1,15)_ = 8.56,* p = *0.01) and a significant interaction between these two main factors (*F*_(1,15)_ = 16.78,* p = *0.001). *Post hoc *tests confirmed that the excitability of the motor cortex induced by MI‐PAS25 was significantly higher than that induced by MI alone (*p = *0.004).

For the nontarget FDI muscle (Figure 4b), the results of a repeated measures ANOVA indicated a significant main effect of time (*F*_(1,15)_ = 6.57, *p = *0.022) but not of interventional protocol (*F*_(1,15)_ = 0.2, *p = *0.661), and the interaction between these factors was not significant (*F*_(1,15)_ = 2.61, *p = *0.127).

#### 3.3.2 Short‐interval intracortical inhibition

**Figure 5:**
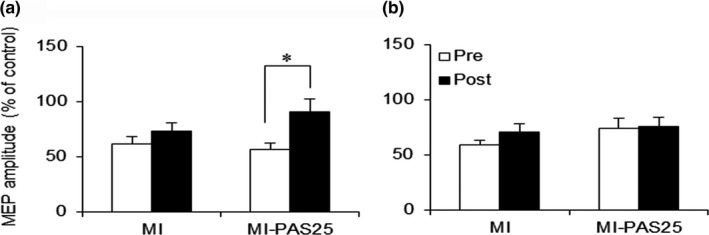
SICI before and after motor imagery (MI) alone or MI‐PAS25. (a) SICI measured immediately after MI‐PAS25 (Post) is significantly smaller relative to that measured before (Pre) MI‐PAS25 in the target APB muscle (*p < 0.05). (b) There is no difference in SICI before and after MI alone or MI‐PAS25 in the nontarget FDI muscle. SICI, short‐interval intracortical inhibition

Figure 5 shows the changes in SICI, as measured by MEP amplitude, induced by MI alone or MI‐PAS25. The results of our ANOVA indicated a significant main effect of time (*F*_(1,15)_ = 10,* p = *0.006). The main effect of the interventional protocol (*F*_(1,15)_ = 0.37,* p = *0.552) and the interaction between interventional protocol and time (*F*_(1,15)_ = 2.65,* p = *0.125) were not significant in the target APB muscle. Paired *t *tests indicated that SICI after the MI‐PAS25 intervention was decreased relative to baseline (*t*_15_ = −2.92, *p* = 0.011), but there was no significant difference in SICI before and after MI alone (*t*_15_ = −1.39, *p* = 0.184).

For the nontarget FDI muscle, the ANOVA results indicated that neither the main effects of the interventional protocol (*F*_(1,15)_ = 1.33,* p* = 0.268) or time (*F*_(1,15)_ = 0.9, *p* = 0.358) nor the interaction between the interventional protocol and time (*F*_(1,15)_ = 0.8,* p* = 0.385) were significant.

### 3.4 Correlation between peak acceleration, corticospinal excitability, and intracortical inhibition

**Figure 6:**
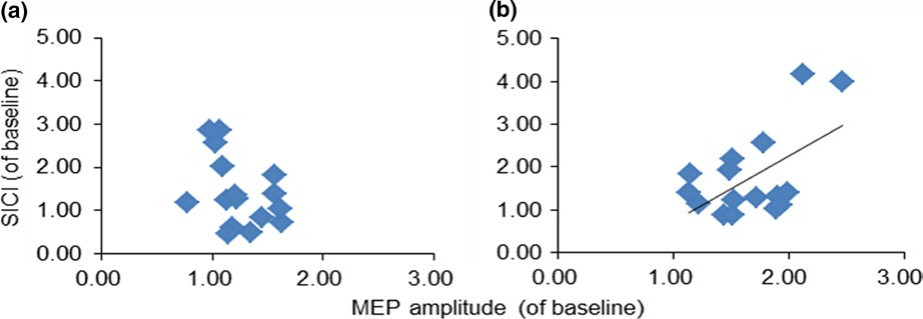
Relationship between corticospinal excitability and intracortical inhibition after MI‐PAS25 or after motor imagery (MI) alone in the APB muscle. The abscissas indicate the MEP amplitudes (of baseline) after MI alone (a) and after MI‐PAS25 (b). The ordinates indicate the SICI (of baseline) after MI alone (a) and after MI‐PAS25 (b). The dark line in (b) is the regression line, suggesting a positive correlation between corticospinal excitability and SICI after MI‐PAS25

We next investigated whether a relationship existed between corticospinal excitability and intracortical inhibition after MI‐PAS25 or after MI alone (Figure 6). The correlation between the MEP amplitude and SICI after MI‐PAS25 intervention was significant (*r = *0.56, *p* = 0.013). However, no significant correlation was detected between corticospinal excitability and intracortical inhibition after MI alone (*r *= −0.4, *p* = 0.062).

**Figure 7:**
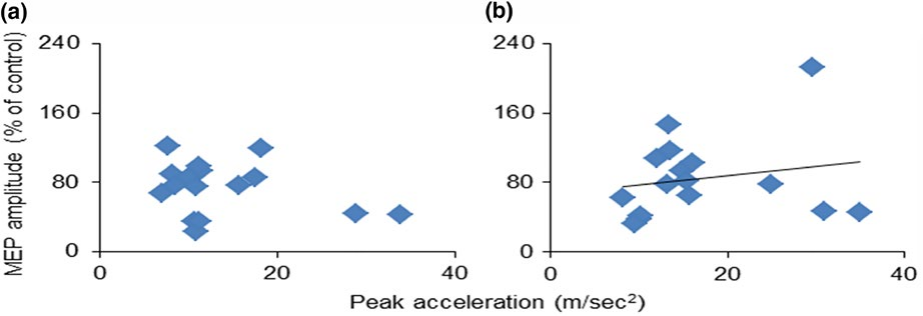
Relationship between peak acceleration and intracortical inhibition (SICI) after MI‐PAS25 or after MI alone. The peak acceleration of the grasping movement after MI alone (a) or after MI‐PAS25 (b) is plotted on the x‐axis, and intracortical inhibition depicted as SICI after MI alone (a) or after MI‐PAS25 (b) is plotted on the y‐axis. Regression analyses (dark line in panel b) indicates a significant positive correlation between peak acceleration and intracortical inhibition after MI‐PAS25, with no significant correlation detected after MI alone

We also investigated whether peak acceleration was associated with intracortical inhibition after MI‐PAS25 or after MI alone (Figure 7). Peak acceleration was significantly correlated with SICI after MI‐PAS25 intervention (*r* = 0.49, *p* = 0.029) but not after MI alone (*r *= −0.28, *p* = 0.147).

## 4 DISCUSSION

In this study, PAS25 was applied to the primary motor cortex immediately after participants performed MI of a grasping movement to test whether cortical excitability or intracortical inhibition change after the noninvasive brain‐stimulated modulation of MI‐enhanced motor learning. We found that although the peak acceleration of the grasping movement was not significantly altered after MI alone, application of PAS25 following MI significantly strengthened motor learning compared with those observed following MI alone. The neurophysiological results showed that after application of MI‐PAS25, corticospinal excitability was significantly increased and SICI was significantly decreased. The decreased SICI was significantly correlated with both peak acceleration and MEP amplitude. In the control experiment, PAS25 alone produced the expected effects of MEP amplitude facilitation in the APB muscle without exerting any significant effect on motor learning at the behavioral level, consistent with the results of previous studies (Jung & Ziemann, 2009; Stefan et al., 2000). Taken together, these results indicate that the change in brain function induced by PAS25 in the human motor cortex enhances the motor learning and corticospinal excitability produced by MI, and that these changes are accompanied by decreased SICI. Thus, we propose that SICI plays an important role in the PAS25 modulation of motor learning.

### 4.1 PAS25 enhancement of MI‐induced motor learning

Improving motor learning by changing brain function is currently a compelling and challenging area of research. In recent years, some scholars have proposed that noninvasive brain stimulation technology improves motor learning (Polania et al., 2018). Studies have shown the efficacy of a device that delivers electricity to the motor cortex, an area of the brain that controls physical skills. One study found that transcranial direct current stimulation (tDCS) of the motor cortex region that controls leg function reduces the perception of fatigue in cyclists, who are then able to pedal longer without feeling tired (Okano et al., 2015). Another study showed that anodal tDCS over the primary motor cortex enhances the effects of MI training on the learning of a finger tapping sequence. A plausible physiological explanation for these effects is that synaptic strength within the primary motor cortex is reinforced by the association of MI training and tDCS (Saimpont et al., 2016). Consistent with the above studies, our study also supports an important role of noninvasive brain stimulation in motor learning. Previous studies have shown that the neural circuits activated by PAS25 are the same as those activated by motor learning (Sale & Mattingley, 2013; Ziemann et al., 2004). We speculate that the neuronal networks activated by PAS25 may be the same as those activated during MI‐induced motor learning. Hence, PAS25 changes the efficacy of the neuronal networks involved in MI processes in the motor cortex and strengthens the learning‐related synaptic connections within this region to promote motor learning.

Another potential explanation for the observed PAS25 enhancement of the learning produced by MI is that it is due to the additive effect of MI and PAS25 on motor learning. However, this explanation is unlikely because in our control experiment, PAS25 alone did not exert any significant increase in motor learning.

### 4.2 Increased corticospinal excitability and decreased SICI after PAS25 modulation of motor learning

The results of this study showed that for the APB muscle, corticospinal excitability after application of MI‐PAS25 was significantly increased relative to that observed after performance of MI alone. Two previous studies have shown that corticospinal excitability induced by PAS25 is increased by concurrent application of MI (Kraus, Naros, Guggenberger, Leao, & Ziemann, 2018; Royter & Gharabaghi, 2016). Although the interstimulus interval between PAS25 and MI differed (i.e., MI followed by PAS25 vs. MI and PAS25 concurrently applied), all interventional protocols produced similar effects through the associative pairing of the different stimuli. Interestingly, whereas motor execution and MI partially activate the same neuronal populations, corticospinal excitability is decreased after motor execution following PAS25 (Ziemann et al., 2004). Thus, although the exact mechanisms still require clarification, the increase in cortical excitability induced by the associative pairing of PAS25 and MI may be attributed to the MI task.

Cortical inhibition is critical for the regulation of neuronal excitability and plasticity (Cash, Jegatheeswaran, Ni, & Chen, 2017). Studies in humans have shown that PAS25 reduces SICI as measured with a threshold tracking method by an amount that is proportional to the size of the PAS25 effect (Murase, Cengiz, & Rothwell, 2015), while engagement of the SICI mediated by GABA_A_ receptors during PAS25 blocks LTP‐like induction in the motor cortex (Elahi, Gunraj, & Chen, 2012). These results are inconsistent with previous results showing that SICI as measured with a standard protocol does not change after PAS25 alone; in addition, we found no previous study describing a correlation between the SICI change and the PAS25 effect (Ni et al., 2014; Stefan et al., 2002). In this study, we found that SICI decreased after PAS25, promoting the motor learning and corticospinal excitability produced by MI. We also found that the decreased SICI was significantly correlated with increased corticospinal excitability and peak acceleration. These findings are consistent with those in human studies showing that cortical inhibition is an important gatekeeper in the induction of cortical plasticity and suggest that decreased SICI may contribute to the enhancement of PAS25 on motor learning (Abraham, 2008; Cash et al., 2017). It is known that MI alone decreases SICI (Takemi, Masakado, Liu, & Ushiba, 2013) and that SICI is further decreased by pairing MI with afferent input (Ridding & Rothwell, 1999). Thus, the additional decrease in SICI observed after MI paired with PAS25 may increase the corticospinal excitability induced by the PAS25 protocol (Kouchtir‐Devanne, Capaday, Cassim, Derambure, & Devanne, 2012).

Short afferent inhibition (SAI) mediated by cholinergic and GABAergic pathways that are activated by afferent input is considered a measure of sensory‐motor interaction (Di Lazzaro, Pilato, Dileone, Tonali, & Ziemann, 2005; Sailer et al., 2003). It is well known that the cholinergic system is involved in learning and memory processes (Bonni, Ponzo, Di Lorenzo, Caltagirone, & Koch, 2017; Turco et al., 2018). Thus, the PAS25 modulation of motor learning induced by MI observed in this study may also be associated with changes in SAI. Furthermore, a previous study showed mutual inhibitory interactions between long‐interval intracortical inhibition mediated by GABA_B_ receptors and SAI (Udupa, Ni, Gunraj, & Chen, 2009). We believe that this inhibitory interaction between SICI and SAI may be involved in the PAS25 modulation of motor learning. However, this hypothesis needs to be tested in future studies. Such an investigation would not only better characterize the neurophysiological underpinnings of motor skills but also may inform future studies to benefit patients with motor impairments caused by stroke.

Our results also showed that there was no change in MEP amplitude or SICI before and after MI‐PAS25 in the nontarget FDI muscle. One previous study found that the enhanced excitability of the APB induced by PAS25 was greater than that of the biceps brachii but not of the abductor digiti minimi. This finding suggests that the motor cortex plasticity induced by PAS25 has a high degree of topographical specificity and requires peripheral median nerve stimuli and TMS to be applied to the same APB muscle (Stefan et al., 2000). Another study showed that although PAS25 targeting the APB muscle enhanced MEP amplitude in the APB and FDI muscles, a priming continuous theta burst stimulation with 150 pulses enhanced the LTP‐like effects induced by PAS25 in the cortical representation of the APB muscle but not in that of the FDI muscle. This finding suggests that heterosynaptic modulation represents a form of plasticity with a high degree of topographic specificity (Ni et al., 2014). Given the findings of both of these aforementioned studies, it is reasonable to postulate that the motor cortex plasticity induced by MI‐PAS25 in this study has a high degree of topographical specificity that requires MI and PAS25 to activate the same APB muscle.

The effects of MI have been examined in the majority of neurophysiological studies during or shortly after the performance of MI (Munzert, Lorey, & Zentgraf, 2009), with relatively few studies investigating the more long‐term aftereffects of MI. Several studies have shown that plastic changes occur in the motor system after a period of MI alone (Meng et al., 2018; Pascual‐Leone et al., 1995). Our results add to this literature, showing that MEPs were enhanced not only after MI alone but also after MI‐PAS25. However, the duration of the aftereffects associated with these interventional protocols warrants additional study because the duration of these aftereffects may significantly impact potential treatments of patients with stroke.

On the basis of all of the aforementioned evidence, we believe that the change in the motor cortex function induced by PAS25 plays an important role in the enforcement of the motor learning produced by MI. Although there is still debate about the peripheral and the central mechanisms that determine motor performance, a nonbrain‐participation model that explains the improvement of motor performance is not feasible in sports physiology (Okano et al., 2015). Therefore, changing brain function using noninvasive brain stimulation technology may be an effective strategy for improving motor learning and when used in sports training, may give athletes a competitive advantage.

In summary, our study showed that PAS25 strengthens the motor learning and corticospinal excitability produced by MI and that this is accompanied by decreased SICI, suggesting that the decreased SICI induced by pairing MI with PAS25 may help to increase motor learning and corticospinal excitability.

## CONFLICT OF INTEREST

The authors have no conflict of interest to declare.
